# Pulsed field ablation using a large-tip focal contact force–sensing catheter with 3-dimensional mapping integration: 12-Month outcomes from the OMNY-AF pilot phase

**DOI:** 10.1016/j.hroo.2026.02.031

**Published:** 2026-03-11

**Authors:** Dinesh Sharma, Moussa Mansour, Aneesh Tolat, Mark Metzl, Christopher Liu, Charles Athill, Luigi Di Biase, Stavros Mountantonakis, Vivek Y. Reddy, Walid Saliba, Andrea Natale, David Newton

**Affiliations:** 1 NCH Rooney Heart Institute, Naples, Florida; 2 Massachusetts General Hospital, Boston, Massachusetts; 3 Hartford HealthCare/University of Connecticut, Hartford, Connecticut; 4 Endeavor Health, Glenview, Illinois; 5 NewYork-Presbyterian Weill Cornell Medicine, New York, New York; 6 San Diego Cardiac Center/Sharp Memorial Hospital, San Diego, California; 7 Montefiore Health System at Albert Einstein College of Medicine, New York, New York; 8 Northwell Health, New Hyde Park, New York; 9 Helmsley Electrophysiology Center, Mount Sinai Fuster Heart Hospital, New York, New York; 10 Cleveland Clinic Foundation, Cleveland, Ohio; 11 Texas Cardiac Arrhythmia Research Foundation, Austin, Texas; 12 Department of Biomedicine and Prevention, Division of Cardiology, University of Tor Vergata, Rome, Italy; 13 Memorial Health University Medical Center, Savannah, Georgia

**Keywords:** Pulsed field ablation, Large-tip focal catheter, OMNYPULSE, PF index, Neurological assessment

**Key Findings**

▪ The OMNY-AF pilot phase showed a strong safety profile, with 0 primary adverse events, 0 magnetic resonance imaging–detected cerebral emboli or lesions, and 0 new or worsening neurological deficits.
▪ High effectiveness was demonstrated, including 100% acute procedure success and 90% freedom from primary effectiveness failure at 12 months.
▪ Electroanatomic mapping integration supported minimal fluoroscopy workflows, with a median fluoroscopy time of 0 minutes and 56.7% of cases performed without fluoroscopy.

Pulsed field ablation (PFA) has emerged as an effective technology for treating atrial fibrillation (AF).^1^^,^^2^ Although periprocedural stroke or transient ischemic attack risk is low, brain magnetic resonance imaging (MRI) studies report 7%–42% rates of silent cerebral events or lesions after catheter ablation.^3^ The large-tip focal PFA platform with contact force (CF)–sensing and pulsed field (PF) index capabilities (OMNYPULSE Catheter and TRUPULSE Generator, Biosense Webster, Inc., part of Johnson & Johnson MedTech, Irvine, CA) has demonstrated favorable 3-month safety and acute efficacy in the first-in-human European study.^4^ However, the technology’s safety and effectiveness results have not been reported in other geographies. Here, we report 12-month outcomes of the pilot phase of the US investigational device exemption study (Figure 1).

**Figure 1:**
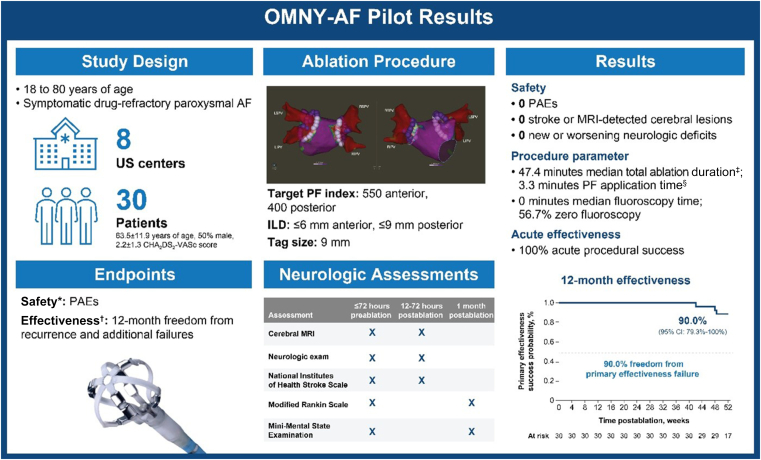
OMNY-AF pilot-phase 12-month results. ∗A PAE is one of the following events that occurs within 7 days (unless otherwise indicated) of ablation: major vascular access complication or bleeding, myocardial infarction, pericarditis, pulmonary edema or respiratory insufficiency, stroke or cerebrovascular accident, transient ischemic attack, thromboembolism, heart block, vagal nerve injury or gastroparesis, permanent phrenic nerve paralysis or diaphragmatic paralysis, cardiac tamponade or perforation (up to 30 days postprocedure), atrioesophageal fistula (up to 90 days postprocedure), PV stenosis (postprocedure), and death (any death within 30 days postprocedure or device-/procedure-related death at any time). ^†^The primary effectiveness endpoint was 12-month freedom from symptomatic and asymptomatic AF/AFL/AT during the effectiveness evaluation period and any of the following failure modes: acute procedure failure, repeat ablation, use of nonstudy catheters, surgical treatment, new or escalation of AADs, or direct current cardioversion. ^‡^*Total ablation duration* is defined as the total duration from the first PF application to the last PF application, including idle time in between. ^§^*PF application time* was defined as the sum of all PF application times, excluding idle time when PF is not applied. AAD = antiarrhythmic drug; AF = atrial fibrillation; AFL = atrial flutter; AT = atrial tachycardia; CHA_2_DS_2_-VASc = congestive heart failure, hypertension, age ≥75 years (doubled), diabetes mellitus, stroke/transient ischemic attack/thromboembolism (doubled), vascular disease, age 65–74 years, sex category; CI = confidence interval; ILD = interlesion distance; LIPV = left inferior pulmonary vein; LSPV = left superior pulmonary vein; MRI = magnetic resonance imaging; PAE = primary adverse event; PF = pulsed field; PFA = pulsed field ablation; PV = pulmonary vein; RIPV = right inferior pulmonary vein; RSPV = right superior pulmonary vein. Images are courtesy of © Biosense Webster, Inc., part of Johnson & Johnson MedTech. All rights reserved.

OMNY-AF (ClinicalTrials.gov identifier NCT06455098), a single-arm, prospective, multicenter study enrolling patients with paroxysmal AF, is being conducted in 2 sequential phases as follows: (1) a pilot phase to assess postablation cerebral emboli with or without neurological symptoms and (2) a pivotal phase to evaluate the protocol-mandated measures of study success. Recommended ablation parameters included the following: 20–50 g CF (from a pressure perspective [force/area], the parameter is equivalent to ∼3–7 g CF with the ThermoCool SmartTouch SF Catheter because of differences in contact surface area); PF index 400 with ≤9 mm interlesion distance (ILD) for the posterior wall, carina, and inferior segments, and PF index 550 with ≤6 mm ILD for the anterior wall, carina, ridge, and superior segments; and 40 mL/min ablation flow rate and 4 mL/min idle flow rate. Patients were followed with 12-lead electrocardiography at 1, 3, 6, and 12 months; 24-hour Holter monitoring at 6 and 12 months, and transtelephonic monitoring weekly from 1 to 5 months and monthly from 6 to 12 months. This study adhered to the principles of the Declaration of Helsinki. Institutional review boards approved the study, and all patients provided consent before enrollment.

The primary safety endpoint was the incidence of primary adverse events (PAEs). Neurological assessments, including cerebral MRI (diffusion-weighted imaging and fluid-attenuated inversion recovery), neurological examination, and standardized scales (National Institutes of Health Stroke Scale, Modified Rankin Scale, and Mini-Mental State Examination) were conducted before and after ablation in all pilot-phase patients at prespecified time points regardless of symptoms. Neurological symptoms were evaluated by site neurologists who were blinded to the cerebral MRI images analyzed by an independent core laboratory. The primary effectiveness endpoint was 12-month freedom from documented (symptomatic/asymptomatic) atrial tachyarrhythmia (AF, atrial flutter, or atrial tachycardia) episodes ≥30 seconds during the effectiveness evaluation period (days 91–365; after a 3-month blanking) and additional failure modes (acute procedure failure, repeat ablation, nonstudy catheter use, surgical treatment, new/escalation of antiarrhythmic drugs [AADs], or direct current cardioversion).

30 patients (mean age 63.5 ± 11.9 years, CHA_2_DS_2_-VASc [congestive heart failure, hypertension, age ≥75 years (doubled), diabetes mellitus, stroke/transient ischemic attack/thromboembolism (doubled), vascular disease, age 65–74 years, sex category] score 2.2 ± 1.3, 50% [15/30] male, 90.0% [27/30] white, 6.7% [2/30] black, 3.3% [1/30] Asian) were enrolled at 8 US centers. Pulmonary vein isolation was performed under general anesthesia. 3 patients received ablation beyond pulmonary vein isolation with the study catheter at the investigator’s discretion, mainly at the posterior wall and roof segments. The median total procedure time was 106.0 (interquartile range 81.0–139.0) minutes, including mandatory preablation mapping with either the investigational catheter or a commercially available high-density mapping catheter and entrance block confirmation with a high-density mapping catheter. The median mapping time, study catheter left atrial dwell time, and ablation time were 6.5 (4.0–11.0), 58.0 (51.0–76.0), and 47.4 (38.9–54.8) minutes, respectively. The total fluoroscopy time was 0 (0–1.6) minutes, with 56.7% of cases (17 of 30) performed with zero fluoroscopy. The CF was 33.1 (25.2–45.3) and 31.0 (22.1–40.8) g in the anterior and posterior regions, respectively. The PF index was 525 (485–550) anteriorly and 464 (424–529) posteriorly. Most targeted veins (95.9% [116 of 121]) were electrically isolated without reconnections after adenosine/isoproterenol challenge without a waiting period. All patients (100%) attained acute procedural success.

No PAEs were observed. No cerebral emboli or lesions were detected by MRI postablation. Neurological examinations indicated no new or worsening deficits. National Institutes of Health Stroke Scale, Modified Rankin Scale, and Mini-Mental State Examination assessment scores improved after ablation, showing no incidence of stroke or cognitive decline.

Freedom from primary effectiveness failure was 90.0% (95% confidence interval 79.3%–100.0%). All 3 primary effectiveness failures were due to arrhythmia recurrences, including 2 asymptomatic AF episodes and 1 symptomatic atrial flutter managed with AADs as the first recurrence failure. Only 1 repeat ablation occurred postblanking. In the repeat procedure, reconnections in the left superior, right superior, and right inferior pulmonary veins were observed; the reconnections may have been due to greater than recommended ILDs, lower CF, and lower PF index in the index procedure. Class I/III AAD use decreased by 86.7% (6.7% [2 of 30] 6–12 months postablation vs 50.0% [15 of 30] at baseline).

In summary, the OMNY-AF pilot phase results demonstrated favorable safety, procedural efficiency, and effectiveness, with no PAEs, cerebral lesions, or neurological deficits, building on the initial safety results reported in the protocol-defined evaluation cohort of the first-in-human study. Pivotal phase results will provide a comprehensive safety and effectiveness profile for this PFA technology.

## Disclosures

Dr Sharma has received grants or contracts from Medtronic; has received consulting fees and support for attending meetings and/or travel from Biosense Webster, Inc., part of Johnson & Johnson MedTech, Boston Scientific, and Medtronic; has participated on a data safety monitoring board or advisory board for the investigator-initiated FOCUS trial; and serves as a board member for the American College of Cardiology Florida Chapter, Arrhythmia Intervention Society, and Naples Comprehensive Health (NCH) Health Care Medical Group. Dr Mansour has received grants or contracts from the POLARIS collaborative study from Biosense Webster, Inc., part of Johnson & Johnson MedTech; has received consulting fees and payment or honoraria for lectures, presentations, speakers bureaus, manuscript writing, or educational events from Abbott, Biosense Webster, Inc., part of Johnson & Johnson MedTech, Boehringer Ingelheim, Boston Scientific, Janssen, Medtronic, Novartis, Pfizer, SentreHEART/AtriCure, and Siemens; and holds equity in EPD-Philips and NewPace Ltd. Dr Tolat has received consulting fees from Medtronic. Dr Metzl has received consulting fees and payment or honoraria for lectures, presentations, speakers bureaus, manuscript writing, or educational events from Atraverse Medical, Boston Scientific, Medtronic, and Philips and holds stock or stock options from Atraverse Medical. Dr Athill has received grants or contracts from Johnson & Johnson; has received consulting fees from Abbott, Biosense Webster, Inc., part of Johnson & Johnson MedTech, and Boston Scientific; and has received payment or honoraria for lectures, presentations, speakers bureaus, manuscript writing, or educational events from Abbott, Biosense Webster, Inc., part of Johnson & Johnson MedTech, and ZOLL. Dr Di Biase has received consulting fees from Biosense Webster, Inc., part of Johnson & Johnson MedTech, Boston Scientific, Abbott, Medtronic, Biotronik, Stereotaxis, ZOLL, Siemens, iRhythm, and Haemodinamics. Dr Mountantonakis has received support for attending meetings and/or travel from Biosense Webster, Inc., part of Johnson & Johnson MedTech. Dr Reddy serves as a consultant for Laminar (including equity stock options). Unrelated to this work, he has served as a consultant for and has equity in Anumana, APN Health, Append Medical, AQUAHeart, AtaCor, Atraverse Medical, Autonomix, BioSig, CardiaCare, CardioFocus, CardioNXT/AFTx, CIRCA Scientific, Conform Medical, CoRISMA, Cortex/Boston Scientific, Corvia Medical, Hangzhou DiNovA EP Technology, East End Medical, ElectroPhysiology Frontiers, Field Medical, Focused Therapeutics, HeartBeam, HRT, InterShunt, Javelin, Kardium, Laminar–Johnson & Johnson MedTech, LuxMed, MedLumics, Orchestra BioMed, PhysioMap, Pulse Biosciences, Restore Medical, Sirona Medical, SoundCath–Boston Scientific, and Volta Medical; has served as a consultant for Abbott, Adagio Medical, AtriAN, BioTel Heart, Biotronik, Boston Scientific, BTL, Cairdac, Cardionomic, Conformal Medical, CoreMap, Fire1, Impulse Dynamics, Medtronic, Novartis, Novo Nordisk, Philips, and SmartValves; and holds equity in DRS Vascular, Manual Surgical Sciences, NewPace Ltd., Nyra Medical, SoundCath, Surecor, and Vizaramed. Dr Saliba has participated in a data safety monitoring board or advisory board for Biosense Webster, Inc., part of Johnson & Johnson MedTech. Dr Natale has received consulting fees from Abbott, Biosense Webster, Inc., part of Johnson & Johnson MedTech, Biotronik, Boston Scientific, and iRhythm and has received support for attending meetings and/or travel from Boston Scientific and Biosense Webster, Inc., part of Johnson & Johnson MedTech. Dr Newton has received consulting fees from AtriCure. Dr Liu has no conflicts of interest.
